# Explainable artificial intelligence based on feature optimization for age at onset prediction of spinocerebellar ataxia type 3

**DOI:** 10.3389/fninf.2022.978630

**Published:** 2022-08-30

**Authors:** Danlei Ru, Jinchen Li, Ouyi Xie, Linliu Peng, Hong Jiang, Rong Qiu

**Affiliations:** ^1^School of Computer Science and Engineering, Central South University, Changsha, Hunan, China; ^2^National Clinical Research Center for Geriatric Disorders, Xiangya Hospital, Central South University, Changsha, Hunan, China; ^3^Center for Medical Genetics and Hunan Key Laboratory of Medical Genetics, School of Life Sciences, Central South University, Changsha, Hunan, China; ^4^Department of Neurology, Xiangya Hospital, Central South University, Changsha, Hunan, China; ^5^School of Basic Medical Science, Central South University, Changsha, Hunan, China; ^6^Key Laboratory of Hunan Province in Neurodegenerative Disorders, Central South University, Changsha, Hunan, China; ^7^Hunan International Scientific and Technological Cooperation Base of Neurodegenerative and Neurogenetic Diseases, Changsha, China

**Keywords:** spinocerebellar ataxia type 3, CAG repeats, age at onset, machine learning, feature optimization, explainable artificial intelligence (XAI)

## Abstract

Existing treatments can only delay the progression of spinocerebellar ataxia type 3/Machado-Joseph disease (SCA3/MJD) after onset, so the prediction of the age at onset (AAO) can facilitate early intervention and follow-up to improve treatment efficacy. The objective of this study was to develop an explainable artificial intelligence (XAI) based on feature optimization to provide an interpretable and more accurate AAO prediction. A total of 1,008 affected SCA3/MJD subjects from mainland China were analyzed. The expanded cytosine-adenine-guanine (CAG) trinucleotide repeats of 10 polyQ-related genes were genotyped and included in related models as potential AAO modifiers. The performance of 4 feature optimization methods and 10 machine learning (ML) algorithms were compared, followed by building the XAI based on the SHapley Additive exPlanations (SHAP). The model constructed with an artificial neural network (ANN) and feature optimization of Crossing-Correlation-StepSVM performed best and achieved a coefficient of determination (R2) of 0.653 and mean absolute error (MAE), root mean square error (RMSE), and median absolute error (MedianAE) of 4.544, 6.090, and 3.236 years, respectively. The XAI explained the predicted results, which suggests that the factors affecting the AAO were complex and associated with gene interactions. An XAI based on feature optimization can improve the accuracy of AAO prediction and provide interpretable and personalized prediction.

## Introduction

Spinocerebellar ataxia type 3 (SCA3), also called Machado–Joseph disease (MJD), is a common polyglutamine (polyQ) disease. PolyQ diseases are neurodegenerative disorders, and include Huntington's disease (HD), spinal bulbar muscular atrophy (SBMA), dentatorubral pallidoluysian atrophy (DRPLA), and spinocerebellar ataxia (SCA) types 1, 2, 3, 6, 7, and 17. The age at onset (AAO) of polyQ diseases is usually at middle age, and after that, the condition progressively worsens for 10–30 years until death (Ross, 1997; Fan et al., 2014). Although many studies have focused on the treatment of polyQ disease, there is no effective clinical treatment (Esteves et al., 2017; Paulson et al., 2017; Coarelli et al., 2018; Brooker et al., 2021; Costa and Maciel, 2022). Current treatments can only alleviate the symptoms and delay the progression of the disease after onset, and the treatment goals are to improve the motor performance and the quality of life (Ashizawa et al., 2018; Rodríguez-Díaz et al., 2018; Klockgether et al., 2019; Lanza et al., 2020). However, patients with mild symptoms are more likely to benefit from treatment (Miyai et al., 2012). Animal experiments also show that some potential treatments may prevent or reverse the progression of the disease, but they are more suitable for the early stage (Ashizawa et al., 2018; Friedrich et al., 2018). Therefore, AAO prediction contributes to early initiation to slow the progression and helps health care and social security agencies provide follow-up visits to improve the treatment efficacy (Jacobi et al., 2015; Paulson et al., 2017).

PolyQ diseases are caused by expanded cytosine-adenine-guanine (CAG) trinucleotide repeats. The relationship between the expanded CAG repeat (CAGexp) length and the AAO of polyQ diseases has been proven, and the AAO decreased with increasing CAGexp length (Collin et al., 1993; Gusella and Macdonald, 2000; Tang et al., 2000; Chattopadhyay et al., 2003; França Jr et al., 2012; Tezenas Du Montcel et al., 2014). However, the relationship between the AAO and modified genes is complex. The CAG repeat length of the expanded *ATXN3* is the major AAO factor of SCA3/MJD, but other polyQ-related genes (*CACNA1A, TBP, KCN3, RAI1, HTT, ATN 1, ATXN1, 2*, and *7*) and gene interactions also have modifying effects on the AAO (Andresen et al., 2007; Tezenas Du Montcel et al., 2014; Chen et al., 2016a). Other polyQ diseases, such as SCA1 (Wang et al., 2019), SCA2 (Hayes et al., 2000; Li et al., 2021), HD (Hmida-Ben Brahim et al., 2014), also have similar relationships.

Models including the maximum likelihood estimation model (França Jr et al., 2012), least-squares linear regression (Collin et al., 1993; Aylward et al., 1996; Peng et al., 2014; Bettencourt et al., 2016), linear regression based on the log-transformed AAO (Andrew et al., 1993; Lucotte et al., 1995; Chattopadhyay et al., 2003), piecewise regression (Andresen et al., 2007), quadratic regression (Tezenas Du Montcel et al., 2014; Chen et al., 2016a), survival models (Brinkman et al., 1997; Langbehn et al., 2004, 2010; Almaguer-Mederos et al., 2010; Du Montcel et al., 2014; De Mattos et al., 2019; Peng et al., 2021b), and machine learning (ML) models (Peng et al., 2021a) have been used for AAO fitting. Most statistical models attempt to investigate the relationship between the AAO and a few modifiers, but only a few studies have focused on AAO prediction, and the prediction accuracy still needs to be improved. For example, previous ML models of AAO prediction found that ML can improve model performance by comparing the performances of linear regression and 6 other ML models, but its overall prediction performance is still limited (Peng et al., 2021a).

The complex relationship between modifiers and the AAO is one reason for inaccurate prediction. Because of the complexity of gene interactions, this study proposes a feature optimization method to better fit non-linear relationships. We tried to use feature crosses to represent gene interactions and then selected the most important features to ensure the efficiency of the models.

Compared with statistical models, ML models address more variables and improve the accuracy, but ML models are black boxes, and it is difficult to explain the prediction results, which limits the application of ML models. Applying explainable artificial intelligence (XAI) in medicine is very important because the lack of interpretability is the reason ML-based clinical decisions are hard to trust and far from clinical practice (Gilpin et al., 2018; Vellido, 2020; Antoniadi et al., 2021; Banegas-Luna et al., 2021). SHapley Additive exPlanations (SHAP) (Lundberg and Lee, 2017) is based on game theory and can provide an interpretation for the output of ML models. We combined SHAP and ML algorithms to build an XAI for AAO prediction.

In this study, we reanalyzed the data from the literature (Peng et al., 2021a), including the largest cohort of Chinese mainland SCA3/MJD populations. This study aims to compare the performance of feature optimization methods and ML algorithms and proposes an XAI for AAO prediction.

## Materials and methods

### Subjects

A total of 1,008 subjects with SCA3/MJD from the Chinese Clinical Research Cooperative Group for Spinocerebellar Ataxias were included in the study. All clinical data of participants were derived from a previous study by Peng et al. (2021a). The AAO was defined as the age at which the first neurological symptoms appeared. This study was approved by the ethics committee of Xiangya Hospital, Central South University, and written informed consent was obtained from all study participants.

### Genotype analysis and statistical analysis

Genomic DNA (gDNA) was extracted from peripheral blood leucocytes using a standard protocol. The CAG repeat sequences of polyQ-related genes (*ATXN3, ATXN1, ATXN2, CACNA1, ATXN7, TBP, HTT, ATN1, KCNN3*, and *RAI1*) were genotyped by polymerase chain reaction and capillary electrophoresis. The sizes of the shorter alleles (A1) and the longer alleles (A2) were considered as different variables, respectively. The relationships between the length of two alleles are also defined as variables, including the mean (M) of the length of two alleles, and the difference (D) of the length of two alleles. In this way, genetic variables about these 10 polyQ-related genes were described as A1, A2, M and D. Candidate predictors included 40 genetic variables. The Pearson correlation coefficient was calculated to evaluate the correlation between the predictors and the AAO.

### Data preprocessing and splitting

We filtered out subjects with *ATXN3* CAGexp repeat lengths less than 60 or higher than 80 because the number of individuals was too small and scattered. Data were randomly divided into a training set and testing set at a ratio of 8:2 based on non-repetitive random sampling. For comparison with the piecewise model proposed in the literature (Peng et al., 2021a), the total testing set was further split into two subsets according to CAGexp repeat length at *ATXN3*.

### Feature optimization

The original feature set included all candidate predictors, including 40 genetic variables. We used 4 different methods to optimize the original feature set.

#### Feature optimization by correlation (feature set 1)

Optimized feature set 1 was selected by a less strict *p*-value (*p* < 0.1) of the Pearson correlation coefficient referring to the methods in the literature (Sun et al., 1996; Hongyue et al., 2017; Angraal et al., 2020; Peng et al., 2021a).

#### Feature optimization by crossing-correlation (feature set 2)

Optimized feature set 2 was optimized by two steps: feature crossing and feature selection based on the Pearson correlation coefficient. Feature crosses were formed by multiplying two features in the original genetic features. After crossing, feature set 2 is then selected by the *p*-value (*p* < 0.01) and *r* value (|r| > 0.2) of the Pearson correlation coefficient.

#### Feature optimization by crossing-correlation-RFE (feature set 3)

Recursive feature elimination (RFE) is a backward feature selection algorithm that removes the least important features from the feature set recursively by training models with different feature sets. Optimized feature set 3 was a subset of feature set 2. After crossing and correlation-based selection, features were selected using an SVM-RFE with 10-fold cross-validation, and the coefficient of determination (R^2^) was used to evaluate the best feature set.

#### Feature optimization by crossing-correlation-stepSVM (feature set 4)

Optimized feature set 4 was also a subset of feature set 2. After crossing and correlation-based selection, features were selected using a StepSVM (Guo and Chou, 2020) with 10-fold cross-validation, and the R^2^ score was used to evaluate the best feature set. A StepSVM is a stepwise method for feature selection based on an SVM. A StepSVM tries every possible subset, including two features, and the subset with the highest accuracy is chosen as the initial feature set. Then, the features were added to the set recursively to achieve a higher accuracy until the accuracy no longer increased or the maximum number of features allowed was reached.

**Figure 1:**
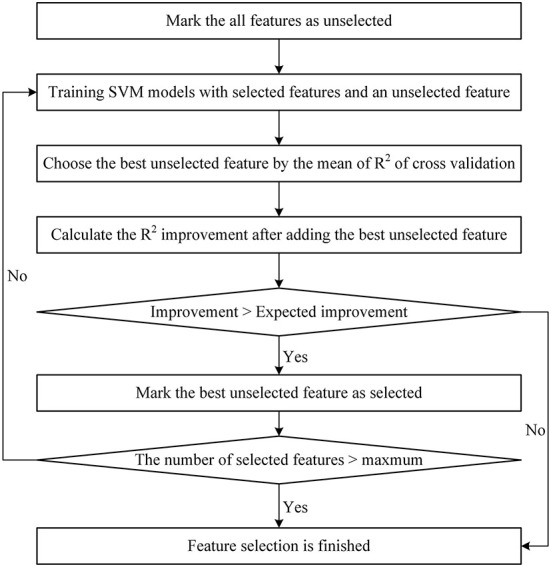
Flowchart of the StepSVM.

Differing from the literature (Guo and Chou, 2020), the StepSVM used in this study started with a feature subset including only one feature. Because (1) there were too many features in the set, the cost of trying every pair of two features is too high; (2) it was confirmed that the CAGexp repeat length is an important feature for AAO prediction. The flowchart of the StepSVM is shown in Figure 1. In this study, the minimum expected R^2^ improvement was set to 0.001, and the maximum number of features selected was set to 30.

### Prediction model construction

We chose 10 ML algorithms for prediction, including linear regression (LR), ridge regression (RR), Lasso, elastic net (EN), Huber regression (HR), K-nearest neighbor (KNN), support vector machine (SVM), random forest (RF), extreme gradient boosting (XGBoost), and artificial neural network (ANN).

We used 4 optimized feature sets and 10 ML algorithms to construct 40 regression models. All models were implemented in Python 3.8 using the scikit-learn (Pedregosa et al., 2011) and XGBoost (Chen and Guestrin, 2016) packages. The parameters of the models were determined by a cross-validated grid search.

#### Linear regression (LR)

LR is a simple multiple regression linear regression model. Usually, the LR coefficient is estimated using the ordinary least squares method, and the objective function of LR is:


(1) $$\begin{array}{c} J\left(\theta \right)=\frac{1}{2}\sum_{i}^{m}{\left(y^{\left(i\right)}\;-\;\theta^{T}x^{\left(i\right)}\right)}^{2} \end{array}$$

#### Ridge regression (RR)

RR is a modified ordinary least squares equal estimate. It gives up the unbiasedness of the least squares and makes the regression process more realistic at the cost of partial accuracy. It can prevent the model from overfitting by adding the L2-norm. The RR objective function is:


(2) $$\begin{array}{c} J\left(\theta \right)=\frac{1}{2}\sum_{i}^{m}{\left(y^{\left(i\right)}\;-\;\theta^{T}x^{\left(i\right)}\right)}^{2}+\text{λ}\sum_{j}^{n}\theta_{j}^{2} \end{array}$$

#### Lasso

Lasso is a linear model that estimates sparse coefficients. Similar to RR, it consists of a linear model and an additional regularization term, but Lasso regression improves the ordinary least squares by adding the L1-norm. The Lasso regression objective function is:


(3) $$J\left(\theta \right)=\frac{1}{2}\sum_{i}^{m}{\left(y^{\left(i\right)}\;-\;\theta^{T}x^{\left(i\right)}\right)}^{2}+\text{λ}\sum_{j}^{n}\left|\theta_{j}\right|$$

#### Elastic net (EN)

EN is a linear regression model applying L1 and L2 regularization. It can not only remove invalid features as Lasso does but also has the stability of RR. The EN objective function is:


(4) $$\begin{array}{c} J\left(\theta \right)=\frac{1}{2}\sum_{i}^{m}{\left(y^{\left(i\right)}\;-\;\theta^{T}x^{\left(i\right)}\right)}^{2}\\ +\text{λ}\left(\rho \sum_{j}^{n}\left|\theta_{j}\right|+\left(1-\rho \right)\text{λ}\sum_{j}^{n}\theta_{j}^{2}\right) \end{array}$$

#### Huber regression (HR)

HR is a kind of robust estimation theory. It reduces the weight of outliers to reduce the impact of outliers on regression results. Its loss combines the advantages of the mean square error (MSE) and the mean absolute error (MAE).


(5) $$\begin{array}{c} J\left(\theta \right)=\sum_{i=1}^{n}\left(\theta +H_{\delta }\left(\frac{S}{\theta }\right)\theta \right) \end{array}$$

(6) $$H_{\delta }\left(S\right)=\left\{ \begin{array}{l} \;\frac{1}{2}{\left(\left|S\right|\right)}^{2},\;for\;|S|\leq \delta \\ \delta |S|-\frac{1}{2}\delta^{2}\;otherwise \end{array}\right.$$

where *θ* is the scale transformation coefficient; *δ* is a threshold, and a smaller δ leads to a solution closer to the MSE, while a larger *δ* leads to a solution closer to the MAE; and S is the residual error between the predicted value and the true value.

#### K-nearest neighbor (KNN)

KNN predicts the label by finding K training samples closest in distance to the new point, and the Euclidean distance is the most common method of distance calculation in KNN. An appropriate value of K is very important for KNN. When K is too small, the prediction result is too sensitive to the nearest point, and when K is too large, distances that are far from the input will also work on the prediction.

#### Support vector machine (SVM)

A SVM performs regression by finding the optimal hyperplane, which divides the data by applying the maximum margin. The maximum margin improves the generalization of the SVM. It has been widely used in disease prediction and has achieved good performance (Chekol and Hagras, 2018; Asha and Vijaya, 2019; Kibtia et al., 2020; Byeon, 2021). The SVM regression function is:


(7) $$\begin{array}{c} J\left(C\right)=C\frac{1}{l}\sum_{i=1}^{l}L_{\varepsilon }\left(y_{i},f\left(x_{i}\right)\right)+\frac{1}{2}{\left\|\omega \right\|}^{2} \end{array}$$

where the coefficients *ω* and *b* are estimated by minimizing the regularized risk function:


(8) $$L_{\varepsilon }\left(y,f\left(x\right)\right)={\left|y-f\left(x\right)\right|}_{\varepsilon }=\max\{0,\left|y-f\left(x\right)\right|-\varepsilon \})\;$$

where $C\frac{1}{l}\overset{l}{\underset{i=1}{\sum }}L_{\varepsilon }\left(y_{i},f\left(x_{i}\right)\right)$ is the empirical error (risk); $\frac{1}{2}||\omega {||}^{2}$ is the regularization term; *L*_ε_(*y, f*(*x*)) is the ε-insensitive loss function; and *C* is a constant regularization parameter, and it is the determinant of the trade-off between deviation and regularization. This indicates tolerance for errors. When *C* is too large, it may cause overfitting; otherwise, it may cause overgeneralization.

#### Random forest (RF)

An RF is an important ensemble model based on bagging. It resamples a dataset by bootstrapping and trains a decision tree on each dataset. The RF prediction result is the combination of the results of the decision trees.

#### Extreme gradient boosting (XGBoost)

XGBoost is a tree-based ensemble model constructed by boosting. It is an improvement of the gradient boosting decision tree (GBDT) algorithm. It sums up the results of many decision trees as the final prediction.


(9) $$\begin{array}{l} J\left(t\right)\;=\sum_{i=1}^{m}L\left(y_{i},{\hat{y}}_{i}^{t}\right)+\sum_{k=1}^{t}\text{Ω}\left(f_{k}\right)\\ \;=\sum_{i=1}^{m}L\left(y_{i},{\hat{y}}_{i}^{t-1}\right)+f_{t}\left(x_{i}\right)+\text{Ω}\left(f_{t}\right)+C \end{array}$$

(10) $$\begin{array}{c} \text{Ω}\left(f\right)=\text{γT}+\frac{1}{2}\text{λ}∥w{∥}^{2} \end{array}$$

where *t* is the number of decision trees; $L\left(y_{i},{ŷ}_{i}^{t}\right)$ is a loss function; and Ω(*f*_*k*_) is the sum of the regularization items of the trees.

#### Artificial neural network (ANN)

An ANN is also called a multi-layer perceptron (MLP). In an ANN, there are multiple hidden layers between the input and output layers. The layers are fully connected by neurons with a non-linear activation function, and the input to each layer is the weighted sum of the output of the previous layer.

In this study, we use a rectified linear unit (ReLU) as an activation function, and it is a piecewise linear function:


(11) $$\begin{array}{r} ReLU\left(x\right)=\max\left(0,x\right) \end{array}$$

The limited-memory Broyden-Fletcher-Goldfarb-Shanno (L-BFGS) algorithm is used to optimize the ANN, which ensures that the network can be optimized quickly and approach the global optimal solution to the greatest extent. The L-BFGS algorithm is an efficient optimization algorithm that can converge faster and perform better on small datasets.

### Evaluation metrics

To evaluate the prediction accuracy and performance of each model, a set of evaluation metrics was used, including the R^2^, mean absolute error (MAE), root mean square error (RMSE), median absolute error (MedianAE), proportion of the samples that |*AAO*_*actual*_−*AAO*_*predicted*_| < 5 (Proportion < 5), and proportion of the samples that |*AAO*_*actual*_−*AAO*_*predicted*_| > 10 (Proportion > 10).

### XAI based on the best ML model

SHAP (Lundberg and Lee, 2017) is based on game theory and uses Shapley values to explain ML models. We chose the AAO prediction model with the best performance and used SHAP as the XAI method to explain the best AAO prediction model on the training set. The explanations provided by XAI include the importance of the features, the impact of features on the model output, and the personalized prediction of each sample.

**Figure 2:**
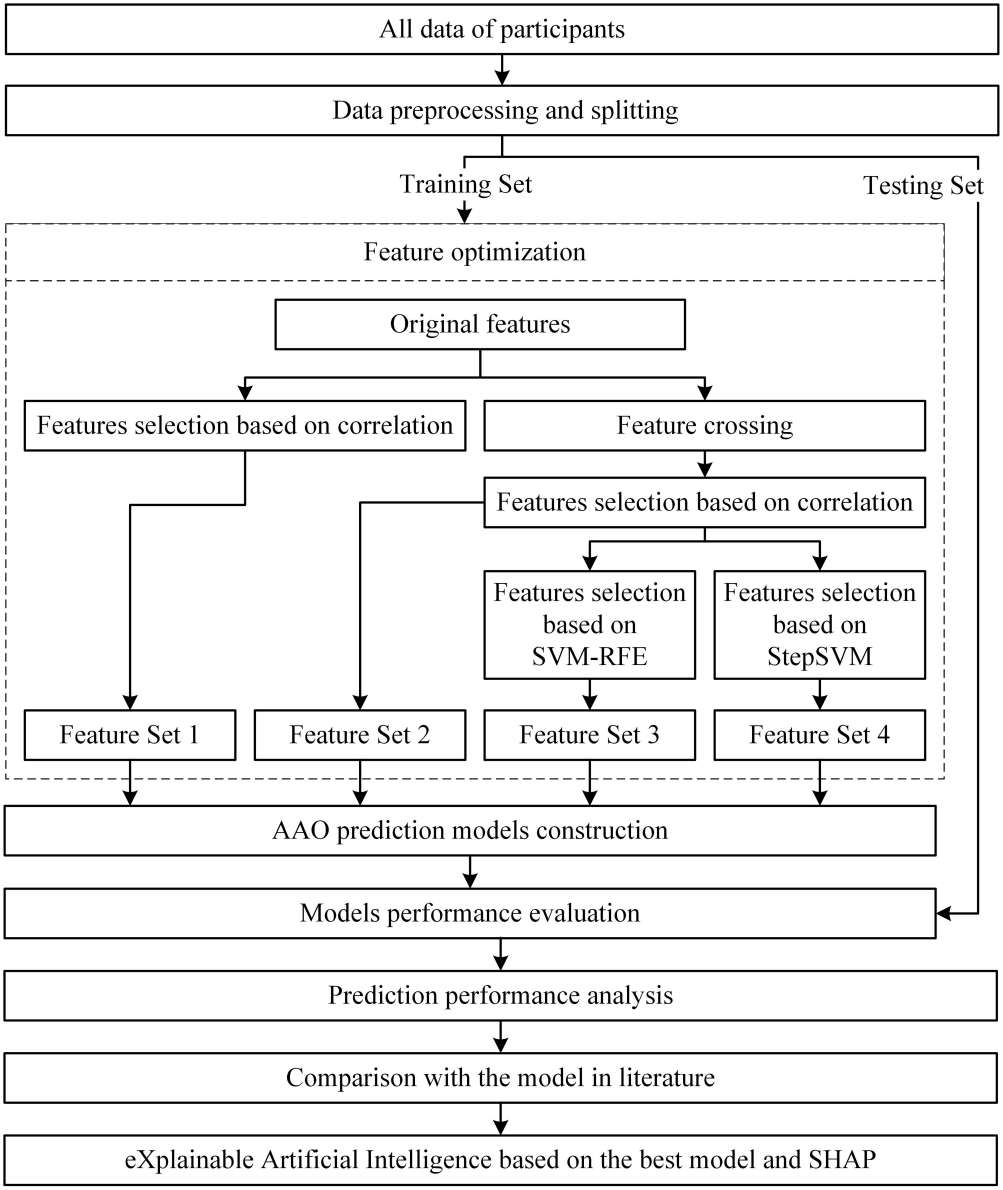
Flowchart of the study.

The flowchart of the process is shown in Figure 2.

## Results

### Description of the subjects and statistical analysis

Among the 1,008 subjects with SCA3/MJD, 549 were male (54.5%), 385 were maternally transmitted, 36 were sporadically transmitted, and 258 were transmission unknown. The mean AAO of all subjects was 35.0 ± 10.2 years, and the mean repeat length of the expanded *ATXN3* allele was 71.8 ± 3.6.

Among the 1,008 subjects, 11 subjects with ATXN3 CAGexp repeat lengths less than 60 or higher than 80 were filtered out. A total of 997 subjects with *ATXN3* repeat lengths between 60 and 80 were used for model construction. There were 797 individuals in the training set and 200 in the testing set. The total testing set was split into two subsets for comparison with the piecewise model proposed in the literature (Peng et al., 2021a). There were 24 individuals in testing subset 1 (*ATXN3* CAGexp ≤ 68) and 176 in testing subset 2 (*ATXN3* CAGexp > 68).

The details of the descriptive statistical analysis are shown in Table 1.

**Table 1:** Results of the descriptive statistical analysis.

**Feature**	**Overall (*****n*** = **997)**		**Training set (*****n*** = **797)**		**Testing set (*****n*** = **200)**	
	**Mean ±SD**	**Min/Max**	**Mean ±SD**	**Min/Max**	**Mean ±SD**	**Min/Max**
ATXN3-A1	19.65 ± 6.24	11/36	19.74 ± 6.27	11/36	19.31 ± 6.08	11/35
ATXN3-A2	71.80 ± 3.32	61/80	71.76 ± 3.30	61/80	71.97 ± 3.39	61/80
ATXN3-D	52.15 ± 7.07	31/68	51.99 ± 7.13	31/68	52.78 ± 6.81	32/66
ATXN3-M	45.73 ± 3.53	37.5/55.5	45.75 ± 3.54	37.5/55.5	45.63 ± 3.51	37.5/54
ATXN1-A1	27.08 ± 1.87	15/31	27.11 ± 1.77	15/31	26.94 ± 2.20	15/31
ATXN1-A2	29.31 ± 1.59	26/39	29.35 ± 1.62	26/39	29.17 ± 1.46	26/33
ATXN1-D	2.24 ± 1.95	0/16	2.24 ± 1.95	0/16	2.23 ± 1.96	0/11
ATXN1-M	28.20 ± 1.43	20.5/33.5	28.23 ± 1.39	23/33.5	28.05 ± 1.59	20.5/31
ATXN2-A1	21.51 ± 1.07	11/22	21.52 ± 1.09	11/22	21.50 ± 0.97	17/22
ATXN2-A2	21.93 ± 1.40	20/33	21.95 ± 1.41	20/31	21.85 ± 1.35	20/33
ATXN2-D	0.42 ± 1.51	0/12	0.44 ± 1.56	0/12	0.31 ± 1.25	0/11
ATXN2-M	21.72 ± 0.97	16/26.5	21.74 ± 1.00	16/26.5	21.63 ± 0.84	19.5/26
CACNA1A-A1	11.38 ± 2.22	4/16	11.36 ± 2.22	4/16	11.46 ± 2.20	5/15
CACNA1A-A2	13.24 ± 1.17	5/17	13.24 ± 1.16	5/17	13.26 ± 1.21	7/17
CACNA1A-D	1.87 ± 2.02	0/11	1.88 ± 2.01	0/11	1.84 ± 2.06	0/9
CACNA1A-M	12.31 ± 1.46	5/16	12.31 ± 1.46	5/16	12.33 ± 1.47	7/15.5
ATXN7-A1	10.29 ± 1.18	6/18	10.30 ± 1.16	6/18	10.23 ± 1.24	6/13
ATXN7-A2	10.97 ± 1.25	7/21	10.95 ± 1.17	7/18	11.04 ± 1.52	8/21
ATXN7-D	0.68 ± 1.14	−2/11	0.65 ± 1.06	−2/6	0.81 ± 1.40	0/11
ATXN7-M	10.63 ± 1.07	7/18	10.62 ± 1.03	7/18	10.66 ± 1.22	7/15.5
TBP-A1	27.68 ± 1.35	20/31	27.69 ± 1.38	20/31	27.63 ± 1.23	21/30
TBP-A2	29.00 ± 1.22	25/35	29.03 ± 1.23	26/35	28.89 ± 1.17	25/34
TBP-D	1.32 ± 1.49	−1/9	1.34 ± 1.49	−1/9	1.23 ± 1.46	0/8
TBP-M	28.34 ± 1.05	23/32.5	28.37 ± 1.08	23/32.5	28.23 ± 0.92	25/30.5
HTT-A1	18.81 ± 1.59	12/25	18.79 ± 1.61	12/25	18.88 ± 1.52	12/23
HTT-A2	20.89 ± 2.21	16/30	20.91 ± 2.25	16/30	20.79 ± 2.00	18/29
HTT-D	2.06 ± 2.14	0/11	2.12 ± 2.16	0/11	1.85 ± 2.05	0/10
HTT-M	19.82 ± 1.57	15/25.5	19.83 ± 1.59	15/25.5	19.80 ± 1.49	16/24.5
ATN1-A1	17.16 ± 2.77	9/24	17.14 ± 2.75	10/24	17.22 ± 2.83	9/22
ATN1-A2	20.46 ± 2.59	12/46	20.39 ± 2.31	12/35	20.72 ± 3.47	14/46
ATN1-D	3.30 ± 2.89	0/28	3.23 ± 2.65	0/15	3.56 ± 3.69	0/28
ATN1-M	18.81 ± 2.26	12/33	18.77 ± 2.17	12/27.5	18.97 ± 2.58	14/33
KCNN3-A1	18.11 ± 1.65	10/21	18.13 ± 1.60	10/21	18.05 ± 1.85	10/20
KCNN3-A2	19.60 ± 1.32	13/30	19.57 ± 1.23	13/30	19.72 ± 1.61	14/30
KCNN3-D	1.51 ± 1.80	0/16	1.47 ± 1.70	0/12	1.66 ± 2.14	0/16
KCNN3-M	19.06 ± 1.22	13/25	19.06 ± 1.17	13/25	19.06 ± 1.40	13/25
RAI1-A1	11.50 ± 0.77	7/13	11.49 ± 0.77	7/13	11.56 ± 0.75	9/13
RAI1-A2	12.11 ± 0.39	10/14	12.10 ± 0.40	10/14	12.13 ± 0.39	10/14
RAI1-D	0.62 ± 0.78	0/5	0.63 ± 0.79	0/5	0.59 ± 0.75	0/3
RAI1-M	11.93 ± 0.52	9/13	11.93 ± 0.53	9/13	11.95 ± 0.52	10/13

### Feature optimization results

#### Feature optimization by correlation (feature set 1)

The results of the Pearson correlation coefficient (Table 2) showed that there were 6 features correlated with the AAO (*p* < 0.05). Because a less strict *p*-value (*p* < 0.1) was applied for feature selection, 8 features were selected for optimized feature set 1, including *ATXN3*-A2, *ATXN3*-D, *ATXN3*-M, *TBP*-A1, *TBP*-M, *ATXN1*-A2, *ATXN2*-A1, and *KCNN3*-D.

**Table 2:** Result of the Pearson correlation coefficient.

**Feature**	***r* value**	***p*-value**	**Feature**	***r* value**	***p*-value**
Sex	−0.0121	0.7340	**TBP-A1**	**−0.0892**	**<0.05**
ATXN3-A1	0.0223	0.5289	TBP-A2	−0.0452	0.2027
**ATXN3-A2**	**−0.7190**	**<0.001**	TBP-D	0.0540	0.1275
**ATXN3-D**	**−0.3554**	**<0.001**	**TBP-M**	**−0.0798**	**<0.05**
**ATXN3-M**	**−0.3164**	**<0.001**	HTT-A1	−0.0055	0.8758
ATXN1-A1	0.0042	0.9061	HTT-A2	0.0180	0.6122
**ATXN1-A2**	**0.0589**	**<0.1**	HTT-D	0.0232	0.5130
ATXN1-D	0.0452	0.2027	HTT-M	0.0092	0.7960
ATXN1-M	0.0370	0.2963	ATN1-A1	0.0403	0.2560
**ATXN2-A1**	**−0.0739**	**<0.05**	ATN1-A2	−0.0168	0.6366
ATXN2-A2	−0.0088	0.8051	ATN1-D	−0.0578	0.1029
ATXN2-D	0.0475	0.1803	ATN1-M	0.0167	0.6385
ATXN2-M	−0.0420	0.2367	KCNN3-A1	0.0479	0.1763
CACNA1A-A1	0.0164	0.6441	KCNN3-A2	−0.0244	0.4910
CACNA1A-A2	0.0205	0.5628	**KCNN3-D**	**−0.0611**	**<0.1**
CACNA1A-D	−0.0130	0.7134	KCNN3-M	0.0064	0.8575
CACNA1A-M	0.0227	0.5220	RAI1-A1	0.0185	0.6029
ATXN7-A1	0.0001	0.9968	RAI1-A2	−0.0285	0.4219
ATXN7-A2	−0.0417	0.2396	RAI1-D	−0.0273	0.4411
ATXN7-D	−0.0453	0.2013	RAI1-M	0.0010	0.9783
ATXN7-M	−0.0174	0.6229			

Bold indicates features optimized through correlation (feature set 1).

#### Feature optimization by crossing-correlation (feature set 2)

After feature crossing, 820 features (780 feature crosses and 40 original features) were used for feature selection. Finally, 75 features were considered to be correlated with the AAO (*p* < 0.01, |r| > 0.2) and included in the optimized feature set 2, which is shown in Table 3.

**Table 3:** Result of feature optimization by cross-correlation (feature set 2).

**Feature**	***r* value**	***p*-value**	**Feature**	***r* value**	***p*-value**
ATXN3-A2	−0.7190	<0.001	ATXN3-A2*CACNA1A-A2	−0.3152	<0.001
ATXN3-D*ATXN3-M	−0.6405	<0.001	ATXN3-D*ATXN1-A2	−0.3099	<0.001
ATXN3-A2*RAI1-A2	−0.6003	<0.001	ATXN3-M*TBP-A1	−0.3081	<0.001
ATXN3-A2*TBP-M	−0.5905	<0.001	ATXN3-M*ATXN2-M	−0.3081	<0.001
ATXN3-A2*TBP-A2	−0.5578	<0.001	ATXN3-D*HTT-A1	−0.3048	<0.001
ATXN3-A2*ATXN3-M	−0.5439	<0.001	ATXN3-M*ATXN2-A1	−0.3039	<0.001
ATXN3-A2*ATXN2-M	−0.5392	<0.001	ATXN3-D*HTT-M	−0.3020	<0.001
ATXN3-A2*TBP-A1	−0.5362	<0.001	ATXN3-M*TBP-A2	−0.3011	<0.001
ATXN3-A2*ATXN2-A1	−0.5250	<0.001	ATXN3-M*RAI1-A2	−0.2995	<0.001
ATXN3-A2*RAI1-M	−0.5224	<0.001	ATXN3-A2*KCNN3-A1	−0.2931	<0.001
ATXN3-A2*ATXN3-D	−0.5006	<0.001	ATXN3-D*CACNA1A-A2	−0.2919	<0.001
ATXN3-A2*ATXN1-M	−0.4763	<0.001	ATXN3-D*ATXN7-A2	−0.2911	<0.001
ATXN3-A2*ATXN2-A2	−0.4454	<0.001	ATXN3-D*ATXN7-M	−0.2882	<0.001
ATXN3-A2*KCNN3-A2	−0.4378	<0.001	ATXN3-A2*ATN1-A2	−0.2849	<0.001
ATXN3-A2*ATXN1-A2	−0.4303	<0.001	ATXN3-D*ATN1-A2	−0.2772	<0.001
ATXN3-A2*KCNN3-M	−0.4238	<0.001	ATXN3-M*RAI1-M	−0.2759	<0.001
ATXN3-A2*ATXN1-A1	−0.4144	<0.001	ATXN3-A2*ATXN7-A1	−0.2711	<0.001
ATXN3-A2*RAI1-A1	−0.4016	<0.001	ATXN3-D*KCNN3-A1	−0.2708	<0.001
ATXN3-D*TBP-M	−0.3632	<0.001	ATXN3-M*ATXN2-A2	−0.2682	<0.001
ATXN3-D*TBP-A1	−0.3629	<0.001	ATXN3-D*ATXN7-A1	−0.2657	<0.001
ATXN3-D	−0.3554	<0.001	ATXN3-D*HTT-A2	−0.2654	<0.001
ATXN3-D*RAI1-A2	−0.3537	<0.001	ATXN3-A2*HTT-A2	−0.2626	<0.001
ATXN3-D*TBP-A2	−0.3536	<0.001	ATXN3-D*ATN1-M	−0.2565	<0.001
ATXN3-D*ATXN2-A1	−0.3524	<0.001	ATXN3-D*CACNA1A-M	−0.2547	<0.001
ATXN3-A2*HTT-M	−0.3475	<0.001	ATXN3-M*KCNN3-A2	−0.2531	<0.001
ATXN3-A2*HTT-A1	−0.3447	<0.001	ATXN3-A2*ATN1-M	−0.2521	<0.001
ATXN3-D*ATXN2-M	−0.3432	<0.001	ATXN3-M*ATXN1-M	−0.2488	<0.001
ATXN3-D*RAI1-M	−0.3380	<0.001	ATXN3-M*KCNN3-M	−0.2450	<0.001
ATXN3-D*KCNN3-A2	−0.3379	<0.001	ATXN3-A2*CACNA1A-M	−0.2397	<0.001
ATXN3-D*ATXN1-M	−0.3266	<0.001	ATXN3-M*ATXN1-A1	−0.2367	<0.001
ATXN3-D*ATXN1-A1	−0.3247	<0.001	ATXN3-M*RAI1-A1	−0.2298	<0.001
ATXN3-A2*ATXN7-M	−0.3213	<0.001	ATXN3-M*ATXN1-A2	−0.2293	<0.001
ATXN3-D*KCNN3-M	−0.3211	<0.001	ATXN3-M*ATXN7-A2	−0.2262	<0.001
ATXN3-D*ATXN2-A2	−0.3182	<0.001	ATXN3-M*ATXN7-M	−0.2187	<0.001
ATXN3-A2*ATXN7-A2	−0.3181	<0.001	ATXN3-A1*ATXN3-D	−0.2133	<0.001
ATXN3-M	−0.3164	<0.001	ATXN3-M*HTT-A1	−0.2133	<0.001
ATXN3-D*RAI1-A1	−0.3162	<0.001	ATXN3-M*HTT-M	−0.2093	<0.001
ATXN3-M*TBP-M	−0.3161	<0.001			

#### Feature optimization by crossing-correlation-RFE (feature set 3)

**Figure 3:**
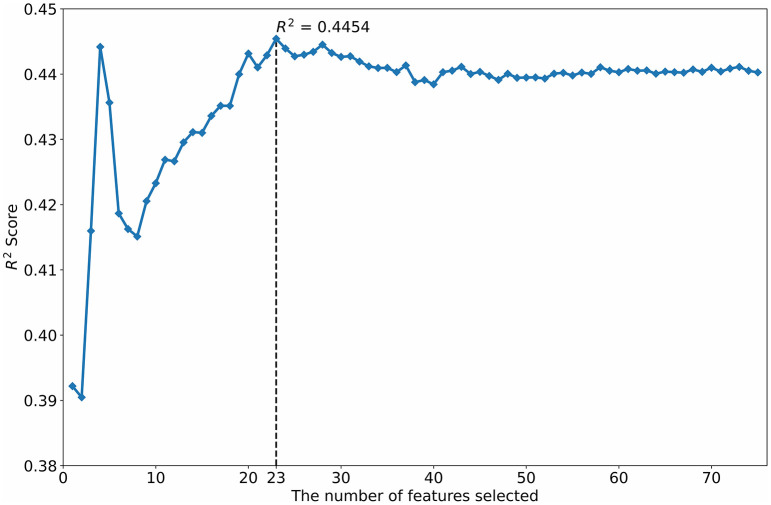
Feature selection using the SVM-RFE.

The R^2^ scores of different numbers of features selected by the SVM-RFE are shown in Figure 3. When the SVM-RFE selected 23 features, the SVM had the highest R^2^ score.

#### Feature optimization by crossing-correlation-stepSVM (feature set 4)

**Figure 4:**
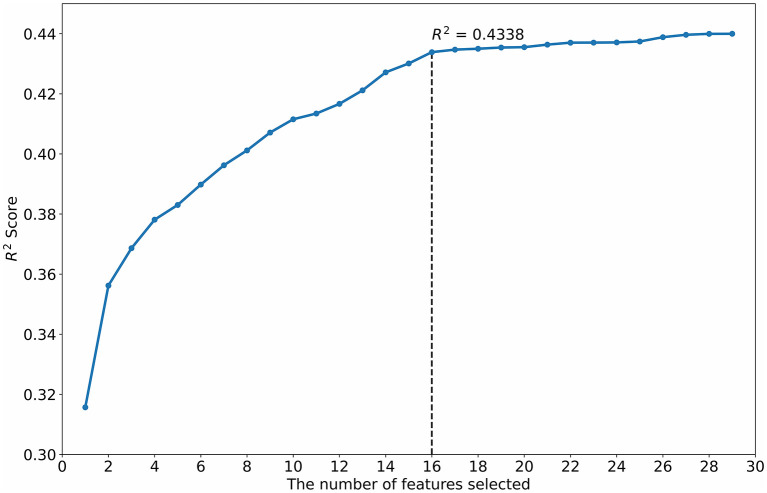
Feature selection using the StepSVM.

The R^2^ scores of the numbers of features selected by the StepSVM are shown in Figure 4. When the number of features selected is greater than 16, the R^2^ improvement is lower than the expected improvement of 0.001.

### Performance of different features and methods

The performance of models constructed with the 4 optimized feature sets and 10 ML methods is shown in Table 4.

**Table 4:** Performances of models constructed with different feature sets and ML methods.

**Method**	**Model**	**R^2^**	**MAE (yrs)**	**RMSE (yrs)**	**MedianAE (yrs)**	**Proportion <5 (no. [%])**	**Proportion > 10 (no. [%])**
LR	1	0.599	4.936	6.546	4.011	126[63.00]	27[13.50]
	2	0.519	5.243	7.166	3.718	124[62.00]	30[15.00]
	3	0.619	4.814	6.380	3.505	129[64.50]	**23[11.50]**
	4	**0.628**	**4.761**	**6.303**	**3.325**	**132[66.00]**	**23[11.50]**
RR	1	0.597	4.972	6.564	3.985	123[61.50]	27[13.50]
	2	0.594	4.955	6.588	3.683	125[62.50]	28[14.00]
	3	0.604	4.908	6.509	3.818	128[64.00]	**25[12.50]**
	4	**0.625**	**4.751**	**6.332**	**3.587**	**134[67.00]**	**25[12.50]**
Lasso	1	0.599	4.936	6.545	4.014	126[63.00]	27[13.50]
	2	0.606	4.902	6.491	3.738	125[62.50]	28[14.00]
	3	0.620	4.801	6.371	3.651	127[63.50]	**24[12.00]**
	4	**0.622**	**4.773**	**6.353**	**3.624**	**133[66.50]**	25[12.50]
EN	1	0.599	4.936	6.546	4.012	126[63.00]	27[13.50]
	2	0.597	4.935	6.565	3.591	126[63.00]	28[14.00]
	3	0.608	4.867	6.471	**3.443**	125[62.50]	25[12.50]
	4	**0.622**	**4.766**	**6.353**	3.639	**132[66.00]**	**24[12.00]**
HR	1	0.609	4.817	6.467	3.540	127[63.50]	26[13.00]
	2	0.605	4.826	6.497	3.548	129[64.50]	29[14.50]
	3	0.620	4.738	6.370	**3.402**	133[66.50]	**24[12.00]**
	4	**0.624**	**4.713**	**6.339**	3.454	**135[67.50]**	**24[12.00]**
KNN	1	0.548	5.474	6.949	4.422	112[56.00]	31[15.50]
	2	0.493	5.743	7.364	4.860	102[51.00]	38[19.00]
	3	0.574	5.191	6.749	4.403	116[58.00]	**25[12.50]**
	4	**0.575**	**5.173**	**6.737**	**4.138**	**117[58.50]**	28[14.00]
SVM	1	0.620	**4.755**	6.375	**3.370**	128[64.00]	24[12.00]
	2	0.594	4.975	6.585	3.655	127[63.50]	25[12.50]
	3	0.616	4.824	6.404	3.490	131[65.50]	**23[11.50]**
	4	**0.621**	4.766	**6.363**	3.698	**134[67.00]**	**23[11.50]**
RF	1	0.622	4.862	6.356	3.746	124[62.00]	23[11.50]
	2	0.618	4.883	6.393	3.863	**128[64.00]**	25[12.50]
	3	0.626	4.863	6.321	3.925	121[60.50]	21[10.50]
	4	**0.629**	**4.815**	**6.300**	**3.858**	125[62.50]	22[11.00]
XGBoost	1	0.598	4.984	6.552	3.807	124[62.00]	**25[12.50]**
	2	0.606	4.881	6.490	3.517	130[65.00]	28[14.00]
	3	0.608	4.963	6.472	3.736	126[63.00]	27[13.50]
	4	**0.623**	**4.773**	**6.345**	**3.462**	**132[66.00]**	27[13.50]
ANN	1	0.614	4.919	6.421	3.790	126[63.00]	25[12.50]
	2	0.624	4.729	6.338	3.582	132[66.00]	21[10.50]
	3	0.639	4.706	6.214	3.551	133[66.50]	26[13.00]
	4	0.653	4.544	6.090	3.236	136[68.00]	22[11.00]

Models 1, 2, 3, and 4 are constructed with feature sets 1, 2, 3, and 4, respectively.

Bold indicates the best result of the models based on the same ML methods.

Underline indicates the best result of all models.

Among the models using different feature sets, the performance of feature set 1 was the worst, which suggests that crossing can improve the prediction accuracy. Models based on optimized feature set 4 have achieved the best prediction, which suggests that the Crossing-Correlation-StepSVM is the best feature optimization method for AAO prediction.

Among the models using different ML methods, models constructed with an ANN perform best. The models constructed with HR also achieve good prediction results.

Among all models, the ANN constructed with feature set 4 (Crossing-Correlation-StepSVM) performs best and achieves the best R^2^ (0.653), MAE (4.544), RMSE (6.090), MedianAE (3.236), and Proportion < 5 (136[68.00]) on the testing set.

### Performance comparison with the model proposed in the literature

The AAO prediction performances on two testing subsets are shown in Table 5. It shows the results of the models constructed with the optimized feature set 4 and results declared in the literature (Peng et al., 2021a).

**Table 5:** Performance on different testing subsets.

**Testing** ** subset**	**Method**	**Model**	**MAE (yrs)**	**RMSE (yrs)**	**MedianAE (yrs)**	**Proportion < 5 (%)**	**Proportion > 10 (%)**
Testing subset 1: *ATXN3* CAGexp ≤ 68	LR	L	6.67	7.83	6.60	38	28
		4	**5.75**	**7.08**	**3.77**	**54**	**17**
	RR	L	-	-	-	-	-
		4	**5.49**	**7.12**	**3.79**	**63**	**21**
	Lasso	L	6.64	7.70	6.53	35	24
		4	**5.49**	**7.18**	**4.05**	63	**21**
	EN	L	6.63	7.70	6.47	35	24
		4	**5.45**	**7.18**	**3.81**	63	**21**
	HR	L	-	-	-	-	-
		4	**5.39**	**7.30**	3.18	63	**17**
	KNN	L	6.41	7.40	6.00	**38**	**17**
		4	**6.03**	**7.02**	**5.35**	**38**	**17**
	SVM	L	7.30	8.57	6.01	34	28
		4	**5.42**	**7.17**	**4.26**	63	**21**
	RF	L	6.74	7.67	6.05	**42**	**17**
		4	**6.15**	**7.30**	**5.31**	**42**	21
	XGBoost	L	**5.56**	**7.13**	**4.15**	**55**	**21**
		4	6.30	7.59	5.16	50	25
	ANN	L	-	-	-	-	-
		4	4.83	5.95	**3.95**	**54**	8
Testing subset 2: *ATXN3* CAGexp >68	LR	L	4.86	6.37	3.75	60	12
		4	**4.63**	**6.19**	**3.30**	**68**	**11**
	RR	L	-	-	-	-	-
		4	**4.65**	**6.22**	**3.59**	**68**	**11**
	Lasso	L	4.82	6.33	3.59	62	12
		4	**4.67**	**6.23**	**3.58**	**67**	**11**
	EN	L	4.80	6.31	**3.63**	63	24
		4	**4.67**	**6.23**	3.64	**66**	**11**
	HR	L	-	-	-	-	-
		4	**4.62**	**6.20**	**3.49**	**68**	**11**
	KNN	L	5.45	6.91	4.70	54	15
		4	**5.06**	**6.70**	**3.76**	**61**	**14**
	SVM	L	4.96	6.47	3.70	60	12
		4	**4.68**	**6.24**	**3.67**	**68**	**10**
	RF	L	4.79	6.34	3.67	**65**	17
		4	**4.63**	**6.15**	**3.48**	**65**	10
	XGBoost	L	4.78	6.31	3.59	65	10
		4	**4.56**	**6.16**	**3.29**	**68**	12
	ANN	L	-	-	-	-	-
		4	4.50	6.11	3.20	70	**11**

Model 4 is constructed with feature set 4.

The results of Model L are those published in the literature.

Bold indicates the best result of models based on the same ML methods.

Underline indicates the best result of all models on the same testing subsets.

In testing subset 1 (*ATXN3* CAGexp ≤ 68), several models achieved a higher proportion < 5 (63%) than the best model, XGBoost, in the literature (55%). HR, as one of the models with the highest proportion < 5 (63%), also achieved the lowest MedianAE (3.18). Additionally, the ANN achieved the lowest MAE (4.83), RMSE (5.95), and proportion > 10 (8%), which showed obvious superiority in prediction accuracy over XGBoost in the literature (MAE: 5.56, RMSE: 7.13, proportion > 10: 21%), but its proportion < 5 (54%) was slightly lower than that of the reported XGBoost model (55%).

In testing subset 2 (*ATXN3* CAGexp > 68), the ANN was the best AAO prediction model, and the evaluated results were MAE (4.50), RMSE (6.11), MedianAE (3.20), Proportion < 5 (70%), which was considerably superior to other models and the previous XGBoost (MAE: 4.78, RMSE: 6.31, MedianAE 3.59: proportion < 5: 65%).

Overall, the results of models constructed with the optimized feature set 4 are better than the results reported in the literature, and the performance of the ANN is the best, followed by HR.

### XAI based on the best ML model

We used the SHAP explainer on the best model (the ANN constructed with the optimized feature set 4) to build the XAI. In the SHAP result, the higher the SHAP value of a feature is, the higher the AAO prediction will be.

#### Importance of features

**Figure 5:**
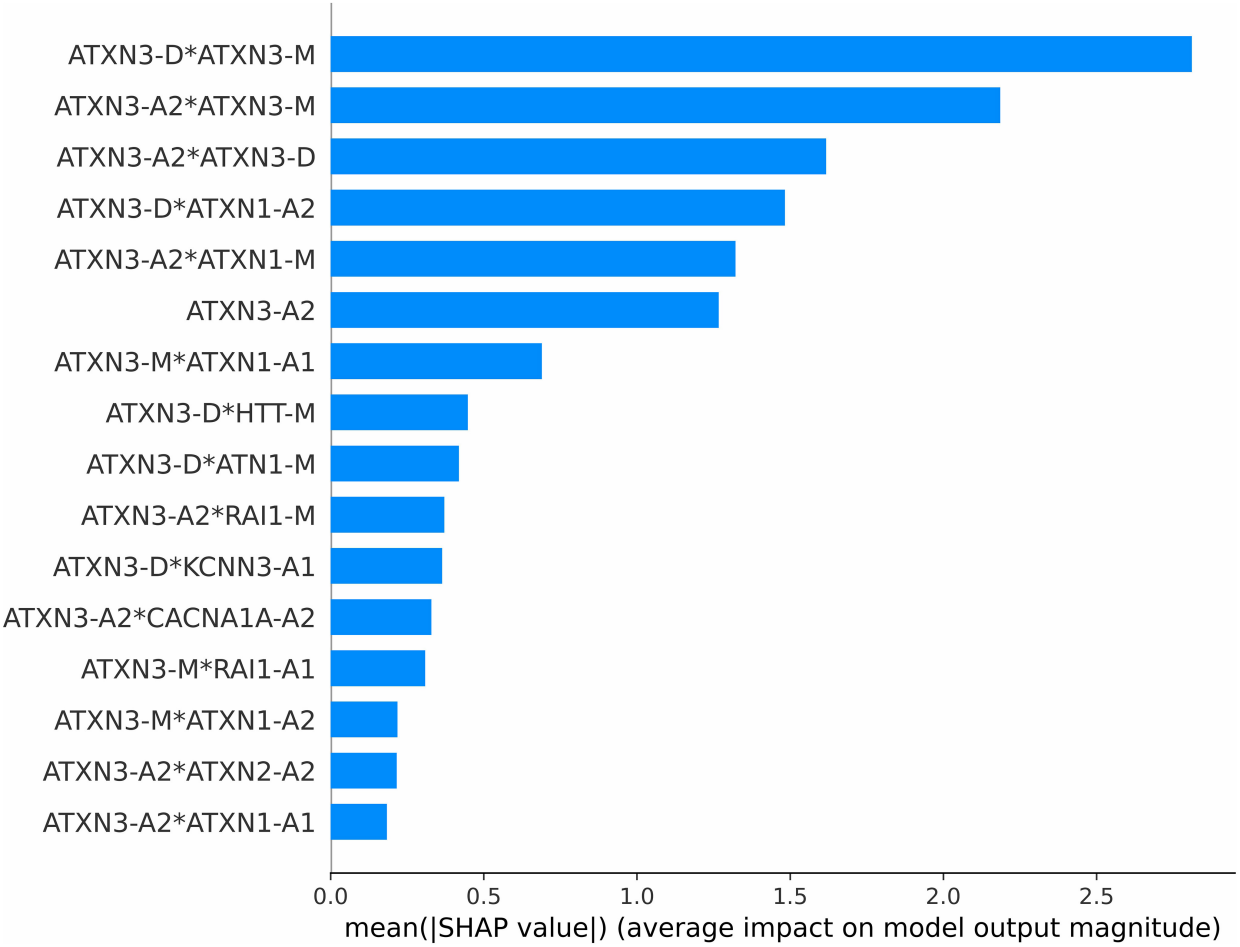
Importance ranking according to SHAP value.

The importance of each feature is measured by the mean of the absolute SHAP value. The importance ranking of the 16 features according to their SHAP values is shown in Figure 5.

The *ATXN3-*related features have the greatest impact on the model, which suggests that *ATXN3-*related features have the greatest impact on the model. The *ATXN*-A2 is not the most important feature, but the three most important features are only associated with the *ATXN3* gene. It suggests that the in addition to the CAG repeat length of *ATXN*, the relationships between the expanded allele and normal allele of *ATXN* cannot be ignored. In addition, the combination of *ATXN3* and *ATXN1* is also very important for AAO prediction.

#### Impact of features on model output

**Figure 6:**
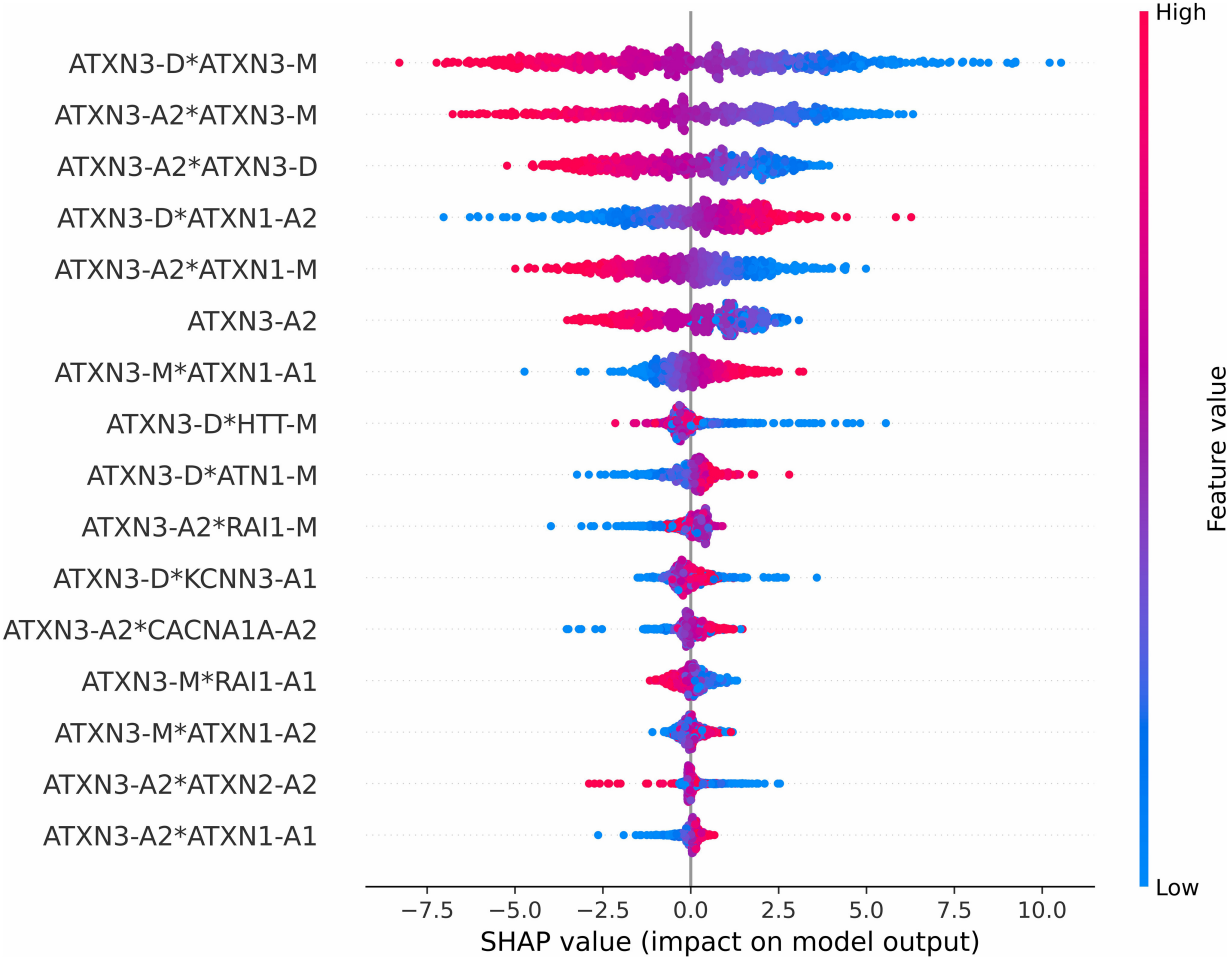
Impact on the model output of all features.

The impact on the model output of all features is shown in Figure 6. The red color represents a higher feature value, while the blue color represents a lower feature value. A positive SHAP value means that the feature results in an increase in the AAO prediction value, and a negative SHAP value means that it leads to a decline. For example, a higher value of the first 3 features will lead to a lower predicted AAO.

**Figure 7:**
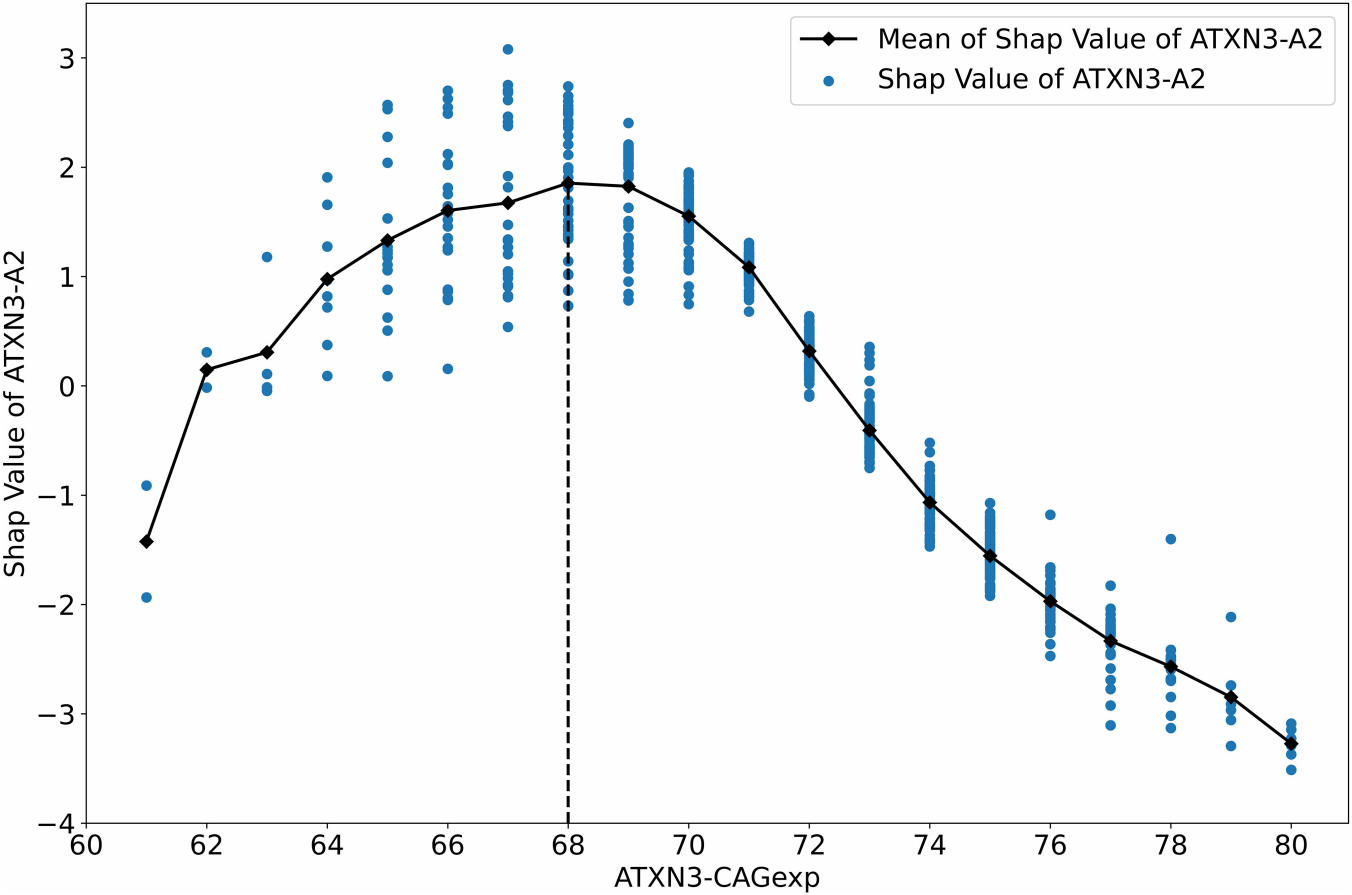
Relationship between the SHAP value and ATXN3 CAGexp.

We used the SHAP value to analyze the impact of *ATXN3*-A2 on AAO output because *ATXN3*-A2 is the only original feature, and it is the most relevant feature to the AAO (*r* = −0.7190, *p* < 0.001). The relationship between the SHAP value of *ATXN3*-A2 and *ATXN3*-A2 before normalization (*ATXN3* CAGexp) is shown in Figure 7. With the increase in the length of *ATXN3* CAGexp, the SHAP value first decreased and then increased, and the segmentation point was at *ATXN3* CAGexp = 68, which is similar to the results of a previous study (Chen et al., 2016b).

**Figure 8:**
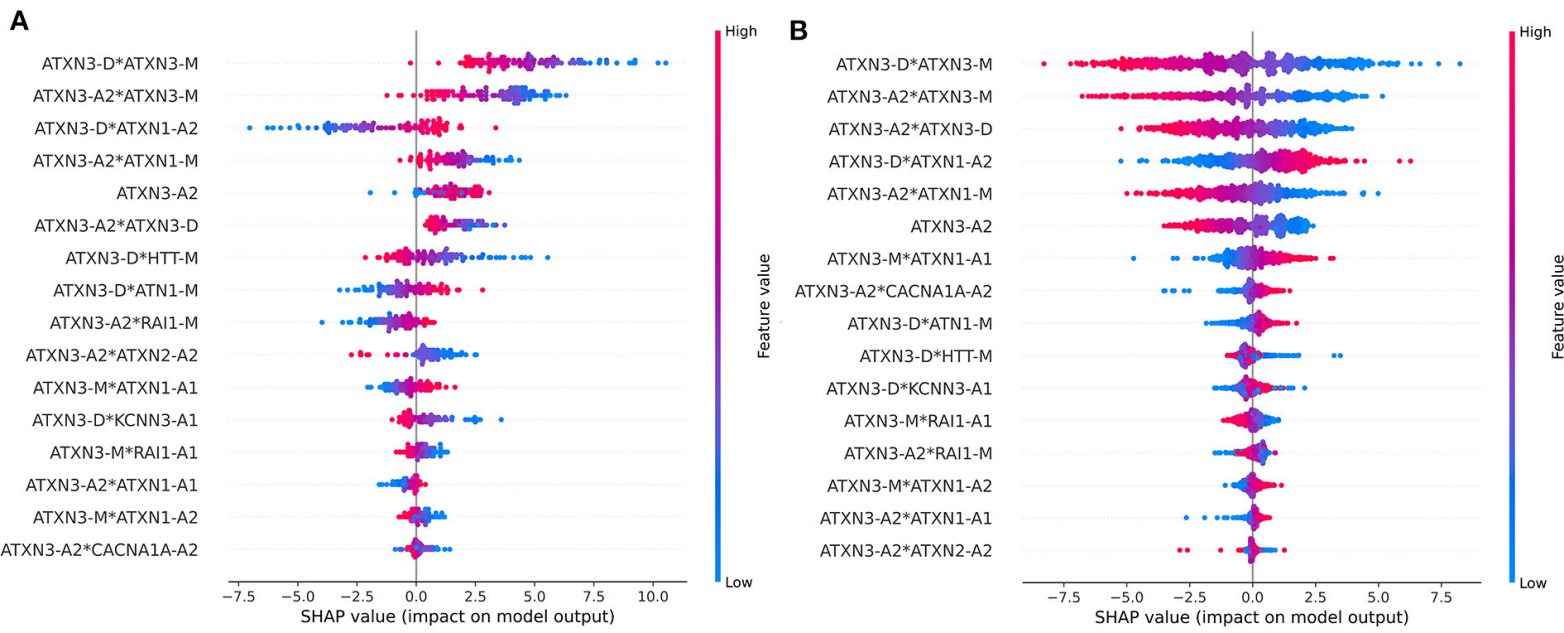
Impact on model output of all features in the two parts of the data: **(A)** ATXN3 CAGexp ≤ 68, **(B)** ATXN3 CAGexp > 68.

The dataset was divided into two parts according to *ATXN3* CAGexp = 68 and the importance of each feature was checked. The ranking of feature importance and the impact of features are different in the two parts of the data is shown in Figure 8. The importance and impact of the features were different in different part of samples. Compared with cases of *ATXN3* CAGexp > 68, the importance of *ATXN3*-related features decreased in cases of *ATXN3* CAGexp ≤ 68.

#### Personalized prediction

**Figure 9:**
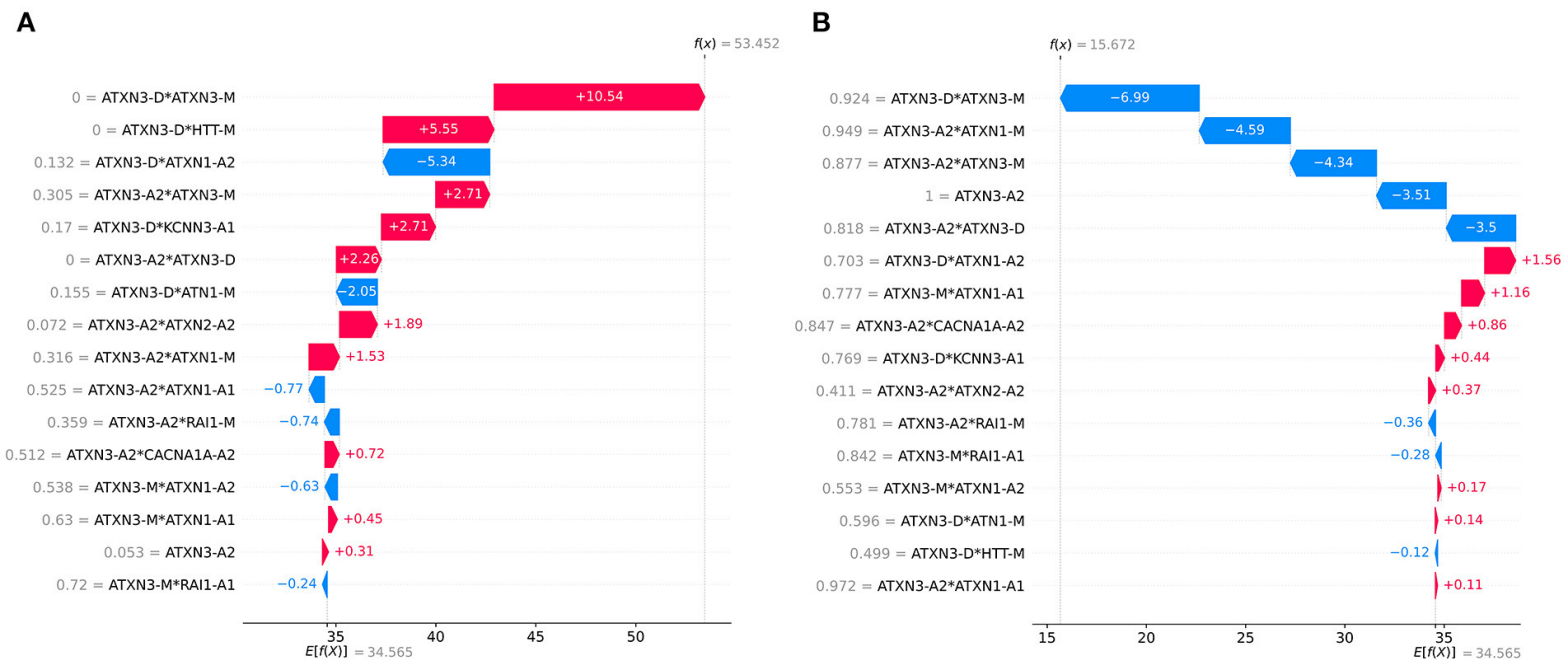
Examples of personalized prediction: **(A)** sample with the largest predicted AAO, **(B)** sample with the smallest predicted AAO. *f* (x) is the predicted AAO and E[*f* (x)] is the expectation of the predicted AAO of all samples.

The SHAP can provide personalized predictions for each sample. Two samples with the largest and smallest predicted AAO were selected as examples of personalized prediction. The relationship between the predicted AAO and features is shown in Figure 9. It explains how the value of each variable leads to the final prediction. The red color represents the increase of AAO prediction, while the blue color represents a decrease of AAO prediction.

## Discussion

To our knowledge, this is the first study attempting to apply XAI to the AAO prediction of SCA3/MJD. This study proposed an XAI model based on feature optimization, which achieved a better AAO prediction accuracy than previous studies (Peng et al., 2021a,b) and can explain the impact of features and provide a personalized prediction.

The relationship between polyQ-related genes and the AAO may be non-linear because of the complex gene interaction. We used feature optimization to model the non-linear relationship between genes and the AAO and compared different feature optimization methods. The best feature optimization method (Crossing-Correlation-StepSVM) can improve the performance of different ML methods. For future research, the feature optimization method can be used for other polyQ diseases because the relationships between the AAO and genetic modifiers are similar in polyQ diseases, such as SCA1 (Wang et al., 2019), SCA2 (Hayes et al., 2000; Li et al., 2021), and HD (Hmida-Ben Brahim et al., 2014).

We compared the performance of 10 ML algorithms for AAO prediction, including LR, RR, Lasso, EN, HR, KNN, SVM, RF, XGBoost, and ANN, and models constructed with the ANN achieved the best accuracy. Additionally, although we chose an ANN to build the XAI model for the final prediction, HR also achieved good results. Combined with feature optimization, HR can provide an effective prediction but requires only a few parameters, which may have more potential in clinical practice.

The XAI proposed in this study performs better in comparison with the results from the literature, and it achieved better R^2^ (0.653), MAE (4.544), RMSE (6.090), MedianAE (3.236), and proportion < 5 (136[68.00]) values. The previous piecewise XGBoost model for AAO prediction of SCA3/MJD patients achieves metrics of MAE (5.56 and 4.78), RMSE (7.13 and 6.31), MedianAE (4.15 and 3.59), and proportion < 5 (55% and 65%) for CAGexp ≤ 68 and CAGexp > 68, respectively (Peng et al., 2021a). Another survival analysis study proposed a parametric survival analysis method to predict the AAO with a reported R^2^ of 0.54 (Peng et al., 2021b).

The interpretability of the XAI model was provided by the result of feature optimization and the SHAP (Figure 5). All optimized features were of the *ATXN3* gene. The most important feature is not *ATXN3*-A2 (the length of *ATXN3* CAGexp) but the combination of *ATXN3*-D (the difference in the length of the two alleles of *ATXN3*) and *ATXN3*-M (the mean length of the two alleles of ATXN3). In addition, optimized features include crosses related to *ATXN1, HTT, ATN1, RAI1, KCNN3, CACNA1A*, and *ATXN2*. The feature crosses related to *ATXN1* are considered of high importance. This suggests that the *ATXN3* gene is the most important AAO modifier, but the relationship between AAO and *ATXN3* is not simply linear, and the factors affecting the AAO are more complex and associated with more modifiers and gene interactions. The proposed XAI also helps to achieve a personalized prediction, which can provide the impact of each feature on the final prediction output in a specific sample (Figure 9). Personalized prediction makes the prediction results of the ML model easier to understand and trust, which may contribute to clinical decisions in the future. For abnormal samples with inaccurate prediction, personalized interpretability can also be combined with medical record analysis, which may also help to discover more AAO modifiers and further improve the prediction model.

This study has some limitations. First, the study reanalyzed data from previous research to provide an XAI model with more accurate prediction but only included data with a small number of subjects from a single center. Second, piecewise models may be more suitable for AAO prediction because the strong negative correlation between the AAO and *ATXN3* CAGexp only exists in cases with *ATXN3* CAG repeats > 68 (Chen et al., 2016b). Our SHAP analysis also supports this segmentation point (Figure 7). However, there are too few cases with *ATXN3* CAG repeats ≤ 68, so we did not use piecewise models as in the literature (Peng et al., 2021a). Third, there are 11 subjects with ATXN3 CAGexp repeat lengths less than 60 or higher than 80 were excluded from our study, while the model in previous literature (Peng et al., 2021a) included all subjects. Comparing with the correlation coefficient reported in the literature, the correlation of *ATXN3*-A2 increased (r_1_ = −0.708, r_2_ = −0.7190) and that of *ATXN3*-D (r_1_ = −0.378, r_2_ = −0.3554) and *ATXN3*-M (r_1_= −0.336, r_2_ = −0.3164) decreased (r_1_ is the Pearson coefficient reported in the literature, r_2_ is the Pearson coefficient in this study). Although the correlation changes are small, there is still the possibility of causing differences in the prediction effect. At last, the improvement of accuracy is still limited, especially for the cases with *ATXN3* CAG repeats ≤ 68. This result suggests a more complex set of AAO modifying factors. There may be other related genes that have not been tested, and environmental influences are difficult to quantify and consider.

## Conclusions

This study proposed an XAI based on feature optimization for AAO prediction in the largest cohort of patients with SCA3/MJD in mainland China. We compared the performance of 4 feature optimization methods and 10 ML algorithms, and the model constructed with the ANN and the feature optimization method of Crossing-Correlation-StepSVM performed best. Then, we built an XAI based on the best model and the SHAP to provide an interpretable and personalized prediction. We hope this study can provide a reference for clinical treatment and help with genetic counseling.

## Data availability statement

The data analyzed in this study is subject to the following licenses/restrictions: datasets analyzed in this study are not publicly available. Further information about the datasets is available to researchers upon reasonable request to the author (HJ). Requests to access these datasets should be directed to HJ, jianghong73868@126.com.

## Ethics statement

The studies involving human participants were reviewed and approved by the Ethics Committee of Xiangya Hospital, Central South University. The patients/participants provided their written informed consent to participate in this study.

## Author contributions

RQ and DR conceived and designed the study. DR and OX did the implementation of the method, conducted the experiments, and generated the results. HJ and LP provided resources and data curation. DR wrote the manuscript. JL, LP, and RQ provided suggestions in writing the manuscript. All authors contributed to the article and approved the submitted version.

## Funding

This study was funded by the National Key Research and Development Program of China (No. 2021YFA0805200 to HJ), the National Natural Science Foundation of China (Nos. 81771231, 81974176, and 82171254 to HJ), the Innovation Research Group Project of Natural Science Foundation of Hunan Province (No. 2020JJ1008 to HJ), the Science and Technology Innovation Group of Hunan Province (No. 2020RC4043 to HJ), the Scientific Research Foundation of Health Commission of Hunan Province (No. B2019183 to HJ), the Key Research and Development Program of Hunan Province (Nos. 2020SK2064 and 2018SK2092 to HJ), the Innovative Research and Development Program of Development and Reform Commission of Hunan Province to HJ, and the Project Program of National Clinical Research Center for Geriatric Disorders (Xiangya Hospital, Nos. 2020LNJJ12 and XYYYJSTG-05 to HJ).

## Conflict of interest

The authors declare that the research was conducted in the absence of any commercial or financial relationships that could be construed as a potential conflict of interest.

## Publisher's note

All claims expressed in this article are solely those of the authors and do not necessarily represent those of their affiliated organizations, or those of the publisher, the editors and the reviewers. Any product that may be evaluated in this article, or claim that may be made by its manufacturer, is not guaranteed or endorsed by the publisher.
