# Supplementary material for: Explainable artificial intelligence based on feature optimization for age at onset prediction of spinocerebellar ataxia type 3
Source: Front Neuroinform. 2022 Aug 30;16:978630. doi: 10.3389/fninf.2022.978630 (PMC9468717; doi:10.3389/fninf.2022.978630)
Supplement: Supplementary file 1 [file Data_Sheet_1.pdf]

Supplementary Material

Glossary

AAO = age at onset;

LR = linear regression;

RR = ridge regression;

EN = Lasso, elastic net;

HR = Huber regression;

KNN = K-nearest neighbor;

SVM = support vector machine;

RF = random forest;

XGBoost = extreme gradient boosting;

ANN = artificial neural network.

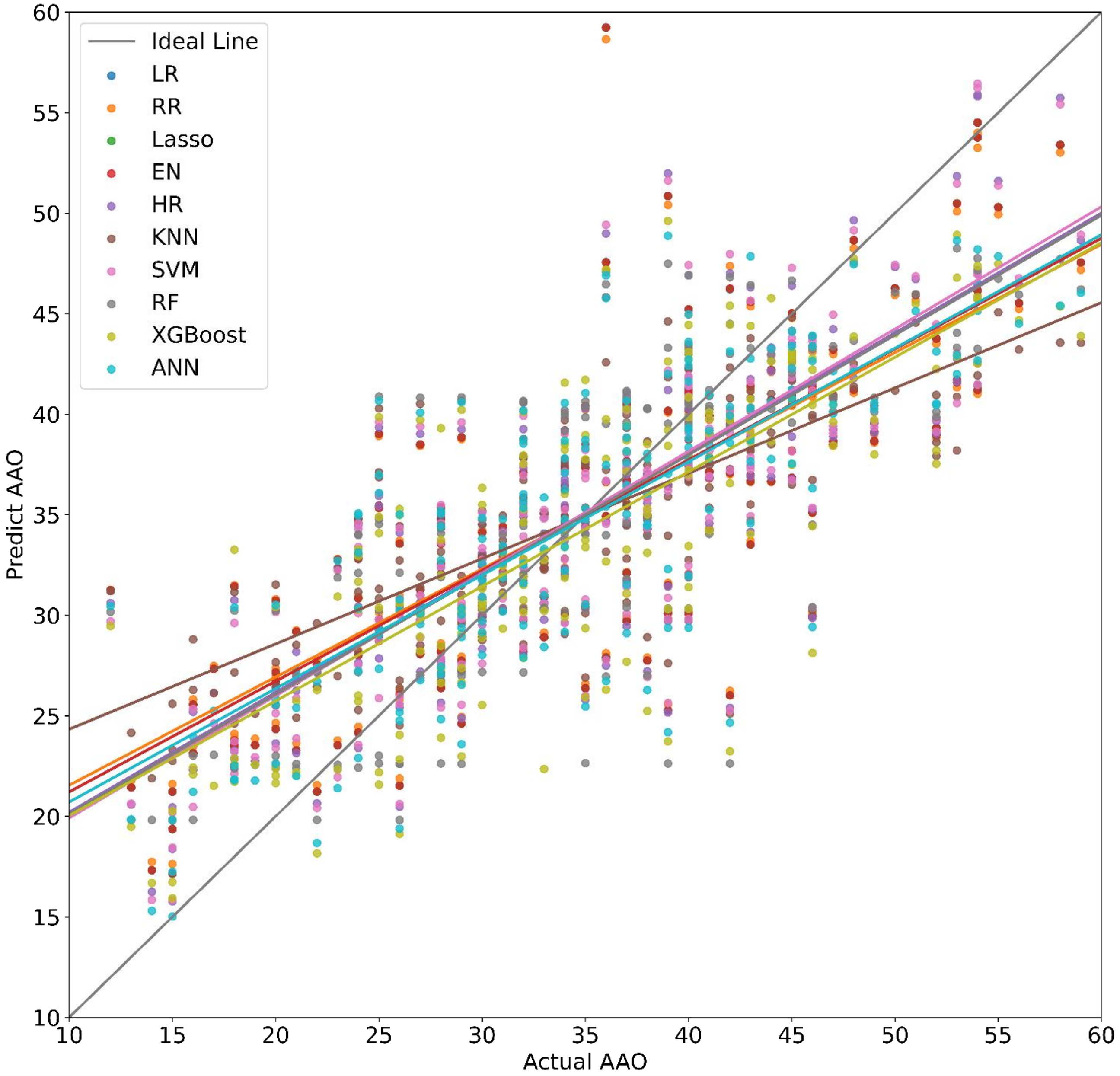

Supplementary Figure 1. AAO prediction results of models constructed with different machine learning algorithms and Feature set 1 in testing set. Dots were plotted for each subject showing predicted AAO (y-axis) versus true AAO (x-axis), with a regression line of optimal fitting of points. HR and RF performed better than other machine learning algorithms.

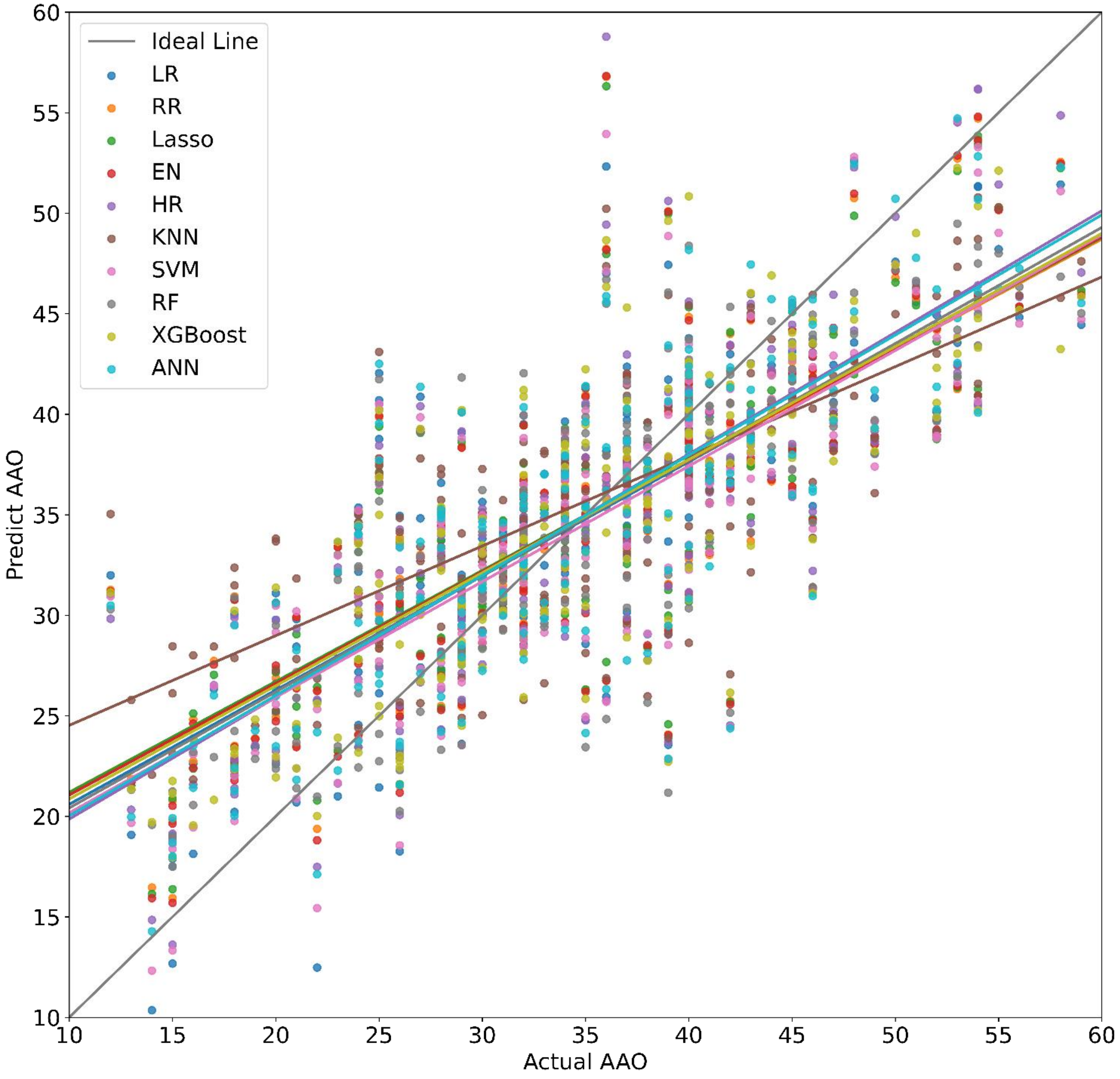

Supplementary Figure 2. AAO prediction results of models constructed with different machine learning algorithms and Feature set 2 in testing set. Dots were plotted for each subject showing predicted AAO (y-axis) versus true AAO (x-axis), with a regression line of optimal fitting of points. HR and ANN performed better than other machine learning algorithms.

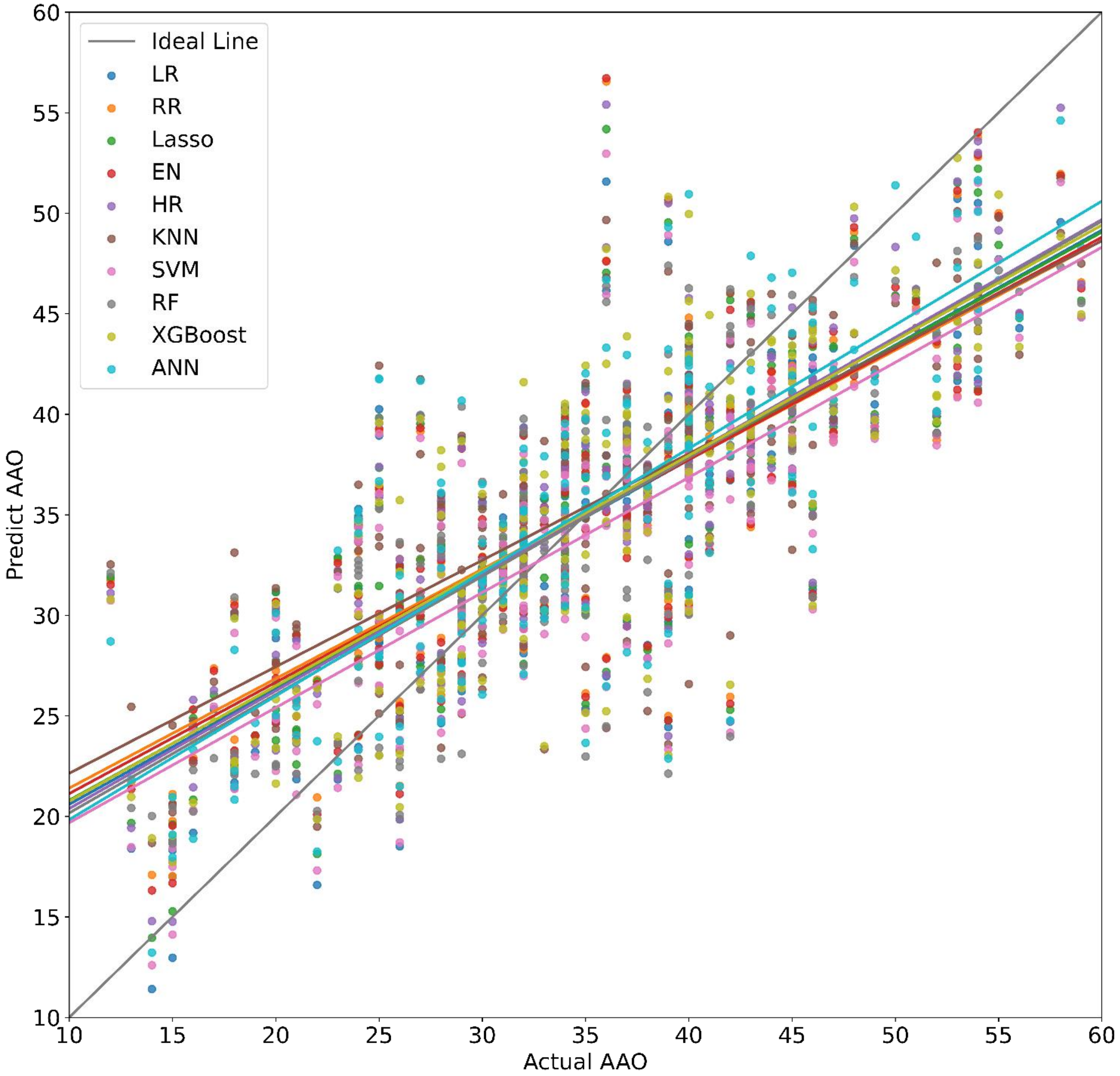

Supplementary Figure 3. AAO prediction results of models constructed with different machine learning algorithms and Feature set 3 in testing set. Dots were plotted for each subject showing predicted AAO (y-axis) versus true AAO (x-axis), with a regression line of optimal fitting of points. ANN performed best in these machine learning algorithms.

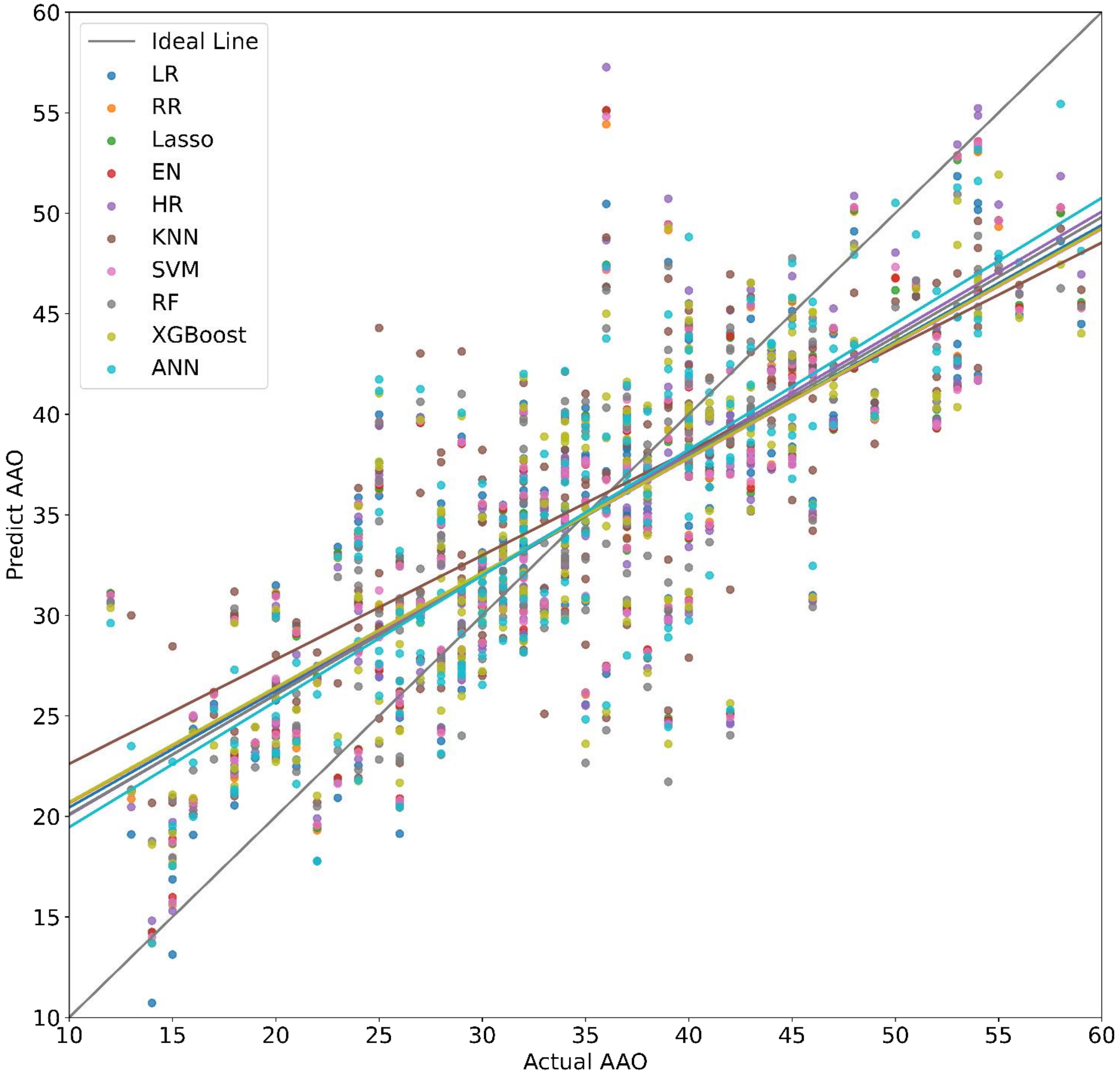

Supplementary Figure 4. AAO prediction results of models constructed with different machine learning algorithms and Feature set 4 in testing set. Dots were plotted for each subject showing predicted AAO (y-axis) versus true AAO (x-axis), with a regression line of optimal fitting of points. ANN performed best in these machine learning algorithms.

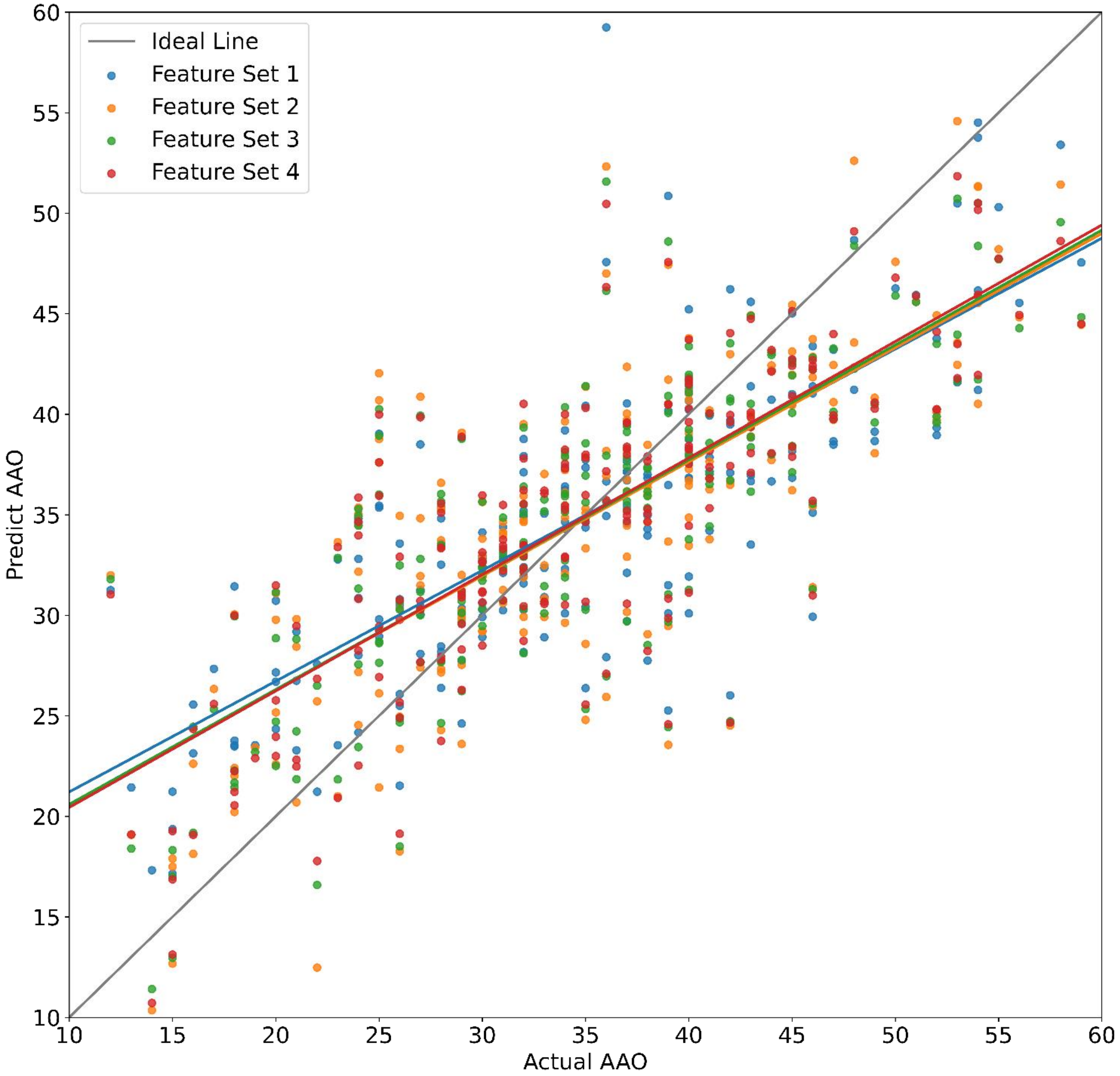

Supplementary Figure 5. AAO prediction results of models constructed with LR and different feature sets in testing set. Dots were plotted for each subject showing predicted AAO (y-axis) versus true AAO (x-axis), with a regression line of optimal fitting of points. The performance of different feature sets is similar.

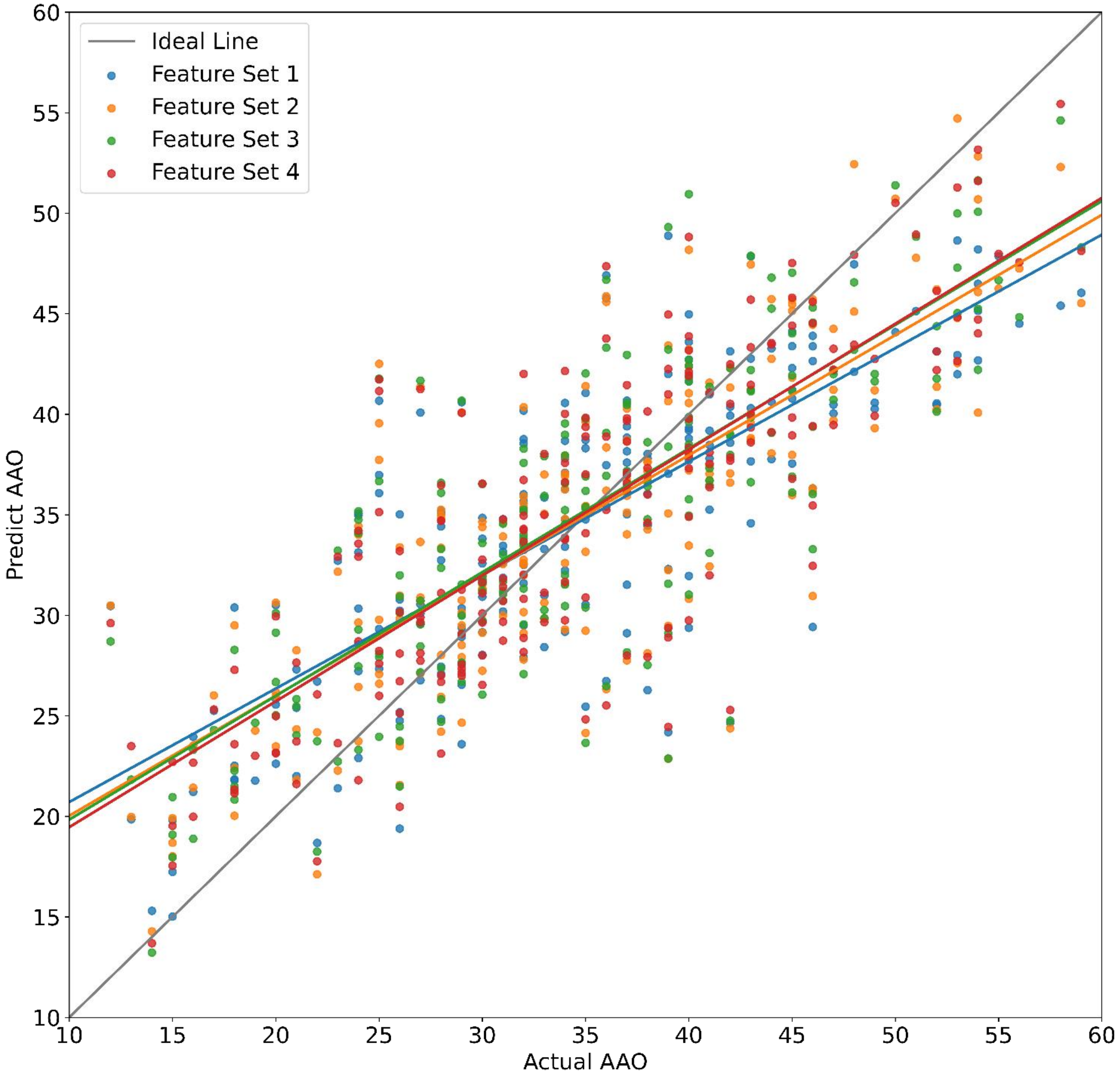

Supplementary Figure 6. AAO prediction results of models constructed with ANN and different feature sets in testing set. Dots were plotted for each subject showing predicted AAO (y-axis) versus true AAO (x-axis), with a regression line of optimal fitting of points. The feature set 4 performed best.
